# The influence of landscape matrix on isolated patch use by wide-ranging animals: conservation lessons for woodland caribou

**DOI:** 10.1002/ece3.695

**Published:** 2013-07-24

**Authors:** Rémi Lesmerises, Jean-Pierre Ouellet, Claude Dussault, Martin-Hugues St-Laurent

**Affiliations:** 1 Département de Biologie, Chimie et Géographie, Université du Québec à Rimouski, Groupe de recherche BORÉAS & Centre d'Études Nordiques300 Allée des Ursulines, Rimouski, QC, G5L 3A1, Canada; 2 Bureau du recteur, Université du Québec à Rimouski300 Allée des Ursulines, Rimouski, QC, G5L 3A1, Canada; 3 Ministère des Ressources naturelles et de la Faune du Québec, Direction de l'expertise Énergie-Faune-Forêts-Mines-Territoire du Saguenay–Lac-Saint-Jean3950 boul. Harvey, 3e étage, Jonquière, QC, G7X 8L6, Canada; 4 Département de Biologie, Chimie et Géographie, Université du Québec à Rimouski, Groupe de recherche BORÉAS, Centre d'Études Nordiques & Centre d'Étude de la Forêt300 Allée des Ursulines, Rimouski, QC, G5L 3A1, Canada

**Keywords:** Anthropogenic disturbances, hurdle model, landscape configuration, range of influence, residual forest patch, space use, surrounding matrix, woodland caribou

## Abstract

For conservation purposes, it is important to design studies that explicitly quantify responses of focal species to different land management scenarios. Here, we propose an approach that combines the influence of landscape matrices with the intrinsic attributes of remaining habitat patches on the space use behavior of woodland caribou (*Rangifer tarandus caribou*), a threatened subspecies of *Rangifer*. We sought to link characteristics of forest remnants and their surrounding environment to caribou use (i.e., occurrence and intensity). We tracked 51 females using GPS telemetry north of the Saguenay River (Québec, Canada) between 2004 and 2010 and documented their use of mature forest remnants ranging between 30 and ∼170 000 ha in a highly managed landscape. Habitat proportion and anthropogenic feature density within incremental buffer zones (from 100 to 7500 m), together with intrinsic residual forest patch characteristics, were linked to caribou GPS location occurrence and density to establish the range of influence of the surrounding matrix. We found that patch size and composition influence caribou occurrence and intensity of use within a patch. Patch size had to reach approximately 270 km^2^ to attain 75% probability of use by caribou. We found that small patches (<100 km^2^) induced concentration of caribou activities that were shown to make them more vulnerable to predation and to act as ecological traps. Woodland caribou clearly need large residual forest patches, embedded in a relatively undisturbed matrix, to achieve low densities as an antipredator strategy. Our patch-based methodological approach, using GPS telemetry data, offers a new perspective of space use behavior of wide-ranging species inhabiting fragmented landscapes and allows us to highlight the impacts of large scale management. Furthermore, our study provides insights that might have important implications for effective caribou conservation and forest management.

## Introduction

**Figure 1:**
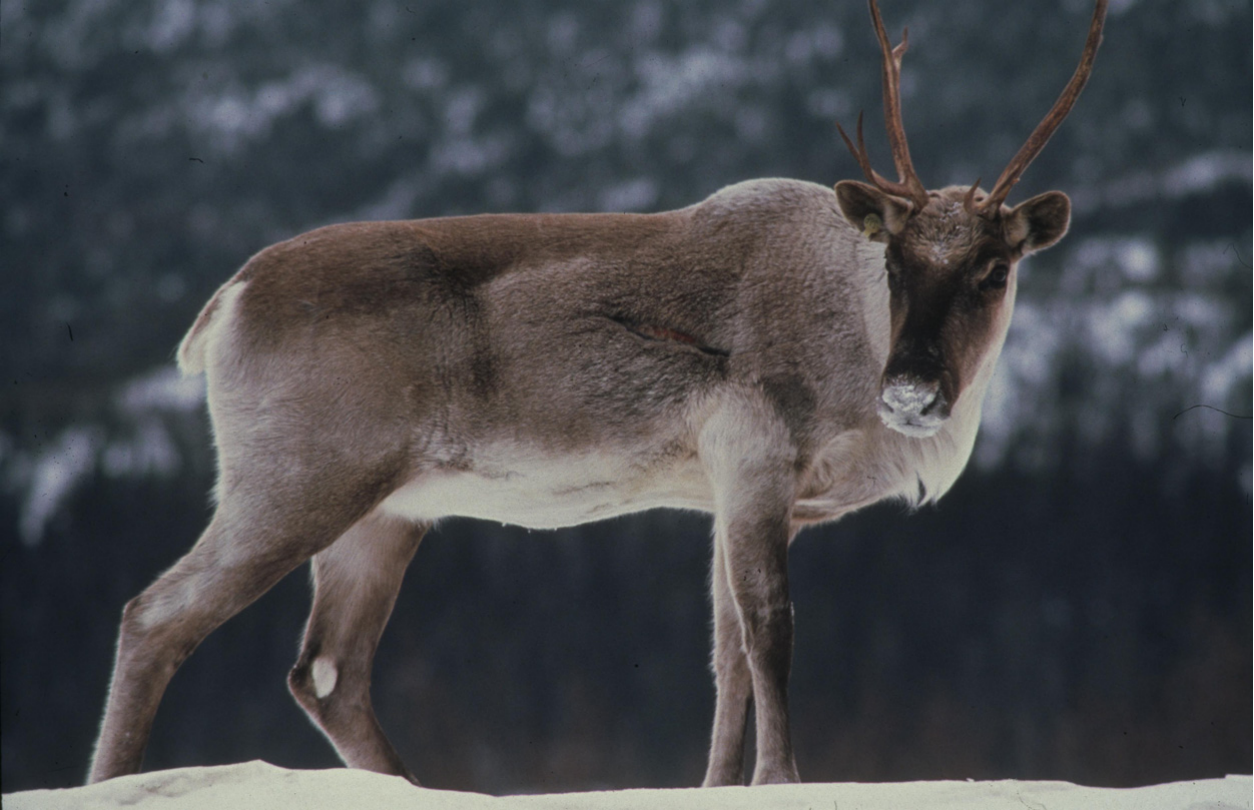
Woodland caribou, Québec, Canada (source: MDDEFP).

Landscape heterogeneity, traditionally due to natural processes, is increasingly an outcome of anthropogenic disturbance regimes, forcing living organisms to adapt to the resulting matrix of habitats (Fischer and Lindenmayer 2007). In the boreal forest biome, natural events like fires and defoliating insect outbreaks have repeatedly created islands of disturbed habitats in a forested matrix (Bergeron et al. 2002). However, the advent of industrial logging and natural resource development has profoundly changed this previous state and mature forests are now becoming isolated in a growing matrix of clear-cuts and early seral stands (Mladenoff et al. 1993). Such a fundamental conversion in forest cover dominance could have important impacts on animal behavior and population dynamics (Andrén 1994), especially for species that are highly mobile and traditionally relied on large, undisturbed habitat patches (Courtois et al. 2007). Species that inhabit old-growth forests, such as woodland caribou *Rangifer tarandus caribou* (hereafter referred to as caribou; Fig. 1), are thought to be more affected by habitat alteration because of their large home range and strict habitat requirements.

Globally, changes in habitat have been identified as the ultimate cause of the recent caribou decline throughout the species historic range (Vors and Boyce 2009). The spatial association between residual mature forests and cutovers or regenerating stands forces caribou to use habitats where predation risk might be higher (Hins et al. 2009; Dussault et al. 2012) and current forest harvesting configuration strategies could be creating ecological traps (Delibes et al. 2001; Battin 2004). Anthropogenic disturbances, including roads, cabins, and industrial sites, are recognized as having a negative influence on caribou and reindeer behavior well beyond the local footprint of these disturbances (Vistnes and Nellemann 2008; DeCesare et al. 2012). These anthropogenic features are having indirect negative influences on caribou habitat because use by humans (e.g., vehicle traffic) may decrease the quality of adjacent environments (Polfus et al. 2011; Leblond et al. 2013), resulting in a global functional loss of suitable habitats.

Many management strategies in North America focus on conservation of large patches of suitable caribou habitat. Recently, Environment Canada (2011) demonstrated that a minimum of ∼65% of a caribou range should be exempt of natural and anthropogenic disturbances' influence to support a self-sustaining population. The federal recovery strategy proposed shortly after (Environment Canada 2012) recognized that fragmentation and spatial configuration of disturbances are important issues to consider, along with the amount of remaining suitable habitat, when assessing the capacity of a caribou range to ensure self-sustainability. However, Environment Canada (2011, 2012) did not provide clear orientations or targets concerning the configuration of disturbances and suitable caribou habitat at the landscape scale. In fact, there is a lack of empirical evidence throughout the caribou literature regarding optimal number, size, composition, and configuration of protected areas, as well as the range of influence of the surrounding habitat matrix. The efficiency of a conservation strategy could be assessed by linking habitat features and disturbances with the space use behavior of the species of concern. Studies interested in quantifying animal space use are usually based on metrics such as home range size, site fidelity and movement rates (Jetz et al. 2004; Faille et al. 2010) or contrast frequented and available habitats to describe habitat selection (Johnson 1980; Manly et al. 2002). All these methods are based on using animals as the sample units, which could limit the establishment of links between animal distribution and landscape structure under a wide range of habitat configurations. For example, two patches of suitable habitat that are of similar size and composition could be frequented in a different manner depending on their shape and surrounding environment (Watling et al. 2011).

Instead of using animal telemetry locations to characterize selection of different habitat types, we propose to focus on habitat patches as the sampling units in order to discriminate factors that might influence their use by a given species. The main advantage of this approach is the possibility to discriminate patch use patterns based on a large set of intrinsic patch characteristics, including the effects of surrounding environment and the scale of its influence. We achieved that using a type of count model (i.e., hurdle model, see below) that allows us to discriminate factors influencing caribou occurrence (presence or absence of an animal within a patch) and intensity of use (number of animal locations in used patches). We were then able to disentangle variables and processes explaining habitat selection patterns at two hierarchical spatial scales (Rettie and Messier 2000; Nielsen et al. 2005). This approach can be implemented easily in a patchy environment where suitable remnants of habitat are surrounded by an unfavorable matrix (Watling et al. 2011). Moreover, this pattern is very likely to apply to an increasing number of species with the upsurge of human activities within prime wildlife habitat (Sanderson et al. 2002) such as boreal forest. We believe that quantifying species use of a given habitat patch is of central importance to direct both landscape management and conservation efforts.

Using a habitat patch framework, our objectives were to (1) determine the extent of mature forest required by caribou following two hierarchical steps; first, where they distribute themselves (i.e., occurrence) and second, how they use the selected patches (i.e., intensity of use). We expected that large patches would have a higher occurrence probability and higher use intensity. We also sought to (2) identify which landscape features influenced the use of these residual forest patches. Knowing caribou generally avoid disturbances, we anticipated large-scale influences of the matrix on patch use. We highlight the relationship between patch size and caribou occurrence and demonstrate the strong influence that human-induced disturbances could have on caribou presence and use of residual forest patches.

## Methods

### Study area

The study area was located north of Saguenay – Lac-Saint-Jean (Québec, Canada) and covered approximately 17 600 km² centered between Piraube Lake in the north (49°42′– 51°00′N, 71°10′– 72°09′W) and Portneuf Lake (48°21′– 49°45′N, 69°51′– 71°12′W) in the south. Forests in the northern part of the study area were characteristic of the spruce *Picea* spp.–moss domain and between 5% (2004) and 10% (2010) of the forest area had been harvested. Hypnaceous mosses with ericaceous shrubs and sparse herbaceous plants were the most common features in the understory, although terrestrial lichen (e.g., *Cladina* spp.) can be locally abundant. Forests in the southern part of the study area were transitional between the spruce–moss domain and the balsam fir *Abies balsamea*–white birch *Betula papyrifera* domain and logging was more common, with ∼35% of the forest area harvested. This study area offers a strong gradient of anthropogenic disturbances from south to north and is representative of boreal forest in Québec, as the spruce–moss domain covers 412 400 km² of the province whereas the balsam fir–white birch domain covers 139 000 km². The mean annual temperature ranges between −2.5 and 0°C and annual precipitation fluctuates between 1000 and 1300 mm, of which 30–35% falls as snow (Robitaille and Saucier 1998). The elevation ranges between 300 and 800 m with low rolling relief.

### GPS telemetry surveys

In order to assess the use of the residual forest patches by caribou, we captured 51 females between 2004 and 2010 (yielding a total of 127 female per year) and fitted them with GPS collars (Lotek Wireless Inc., Newmarket, ON, Canada, models 2200L and 3300L; Telonics Inc., Mesa, AZ, USA, models TGW3600 and TGW4600). Captures and manipulations were approved by the Animal Welfare Committee of the Université du Québec à Rimouski (certificate #36-08-67). Only females were collared because their behavior is likely to influence calf survival (Dussault et al. 2012), and that female survival was shown to have more influence on ungulate population dynamics than sex ratio (Solberg et al. 1999; Gaillard et al. 2000). We nevertheless recognized that behavior could differ between males and females so our inferences should be limited to females only. As GPS collars were programmed to attempt a location every 1–4 h depending on the year and individual, we systematically subsampled our data set to retain one location per individual every 4 h. The study area was delineated for each year using a 100% minimum convex polygon (MCP) using all locations of all animals (second-order habitat selection; Johnson 1980). We conducted our analyses by biological periods (spring: April 15th to May 20th; calving: May 21st to June 20th; summer: June 21st to September 14th; rut: September 15th to October 31st; winter: November 1st to April 14th) because caribou habitat requirements and selection patterns differ temporally (Hins et al. 2009).

### Statistical approach

Our analyses used the residual forest patch as the sampling unit in order to identify which variables (residual patch size, forest type inside patch and proportion of habitat types altogether with cabin, and road density around patch) could explain patch use by caribou. We achieved that using hurdle models (i.e., a type of count model; Zeileis et al. 2008) instead of a more commonly used habitat selection method (e.g., resource selection functions, RSF; Manly et al. 2002) because they gave us a precise description of the intrinsic characteristics of residual patches that are used by caribou while considering the caribou's scale of response to the surrounding environment. This two-component analysis allowed us to identify the variables explaining caribou occurrence (i.e., presence or absence of an animal within a patch) and, secondly, intensity of use (i.e., number of animal locations within used patches). Such analytical framework offered us the opportunity to highlight contrasted patterns of patch use that could differ between two hierarchical scales, for example, if a given habitat has a positive effect on occurrence but a negative influence on the amount of time spent within the selected patches (i.e., intensity of use).

#### Residual patch delineation

In order to delineate residual forest patches, we used ecoforest maps provided by the Ministère des Ressources naturelles et de la Faune du Québec, which are updated each year with new natural and anthropogenic disturbance polygons (e.g., forest fires, cutblocks, windthrows). Minimum mapping unit size was 4 ha for forested polygons and 2 ha for nonforested areas (e.g., water bodies). We classified forest stands into categories relevant for caribou ecology based on studies of their habitat selection in Québec (Courtois et al. 2007; Hins et al. 2009). Since mature coniferous forests (≥75 % of conifers) and open lichen woodlands are known to be strongly selected by caribou almost year-round (Moreau et al. 2012), we used only stands ≥50 years old to delimit residual patches. We also included deciduous and mixed stands (>25 % of deciduous species) of the same age class to determine their influence on caribou space use, as they are often interspersed with coniferous forest and thus included in protected areas when present in limited amounts (Courtois et al. 2007). Because residual forests are often distributed in linear strips (<120 m wide) between adjacent cutblocks according to provincial forestry regulations (Hins et al. 2009), most of the mature forest appears physically connected. In order to disconnect the residual forest fragments that are linked by these narrow strips, we implemented a negative buffer of 60 m to remove residual forest strips and thereafter removed all residual fragments smaller than 2 ha in size. We then applied a positive buffer of 60 m to restore the original size of remaining patches and obtained patches ranging from 4 to ∼170 000 ha. There is an inherent trend in this approach, even if locations are distributed at random; large patches have higher probability of being used and when considering only used patches, larger ones are more likely to support lower location densities. We adjust for this inherent bias by drawing a random number of points equal to the number of real caribou locations inside our study area and identifying the patch size at which random point density decreased to the mean observed for the entire study area. This minimum patch size was 30 ha; all smaller patches were then removed from the sample and diluted into the matrix. Of the remaining habitat (i.e., the matrix), we considered only habitat types expected to influence patch use by caribou (Table 1).

**Table 1:** Description of variables (mean ± standard error) used to model caribou presence and intensity of use within residual forest patches in the Saguenay – Lac-Saint-Jean region, Québec, Canada (2004–2010; *n* = 3744)

Variable	Description	Mean (±SE)
Residual forest patch attributes		
Area	Area (in ha)	585 (±6403)
Mixed	Proportion of mixed and deciduous stands >40 years old	0.06 (±0.07)
Buffer zone attributes (in 7500 m radius)		
Cut	Proportion of cutblocks ≤20 years old	0.20 (±0.15)
Open	Proportion of open areas originating from both natural and anthropogenic disturbances >20 years old	0.04 (±0.08)
Regen	Proportion of stands >20 and ≤40 years old	0.25 (±0.16)
Conifer	Proportion of coniferous stands >40 years old	0.29 (±0.13)
Wetland	Proportion of wetlands	0.02 (±0.02)
Lichen	Proportion of open lichen woodlands	0.01 (±0.02)
Cabin	Density of cabins and industrial sites (nb/km^2^)	0.30 (±0.28)
Road	Density of roads (km/km^2^)	1.71 (±0.68)

#### Scale-sensitive effects of the surrounding environment

We tested whether landscape features in the adjacent matrix surrounding the residual forest patches could influence caribou patch use; we then aimed to delineate the range of influence of different landscape features on caribou behavior. To do so, we used a multiscale approach and calculated the proportion (or density) of seven variables (Table 1) around each residual forest patch within incremental radius buffers of 100, 200, 300, 400, 500, 750, 1000, 2000, 3000, 4000, 5000, and 7500 m. These buffer radii are based on the maximal avoidance distance suggested by Vistnes and Nellemann (2008) for reindeer and caribou. We determined the best scale for each variable using Akaike's Information Criterion (AIC) and conducted the analyses independently for each period, as we expected varying responses throughout the caribou annual cycle. For each variable, we used the best range of influence (i.e., buffer size) for subsequent analyses (see Leblond et al. 2011 for a similar approach). All geomatic analyses were carried out using ArcGIS 9.3.1 (ESRI 2009).

#### Model selection and statistical analyses

We developed a set of six candidate models (Table 2). Model 1 only included the year and the area of residual forest patches; these two variables were included in all models to control for their effects. Model 2 accounted for intrapatch composition, whereas Model 3 included variables based on the hypothesis that the environment surrounding a patch is more important than the patch composition. Model 4 was similar to Model 3 but included perennial disturbances (roads and cabins). Model 5 considered perennial disturbances surrounding the patch only and intrapatch composition and finally, Model 6 included all variables. We log transformed patch area to allow model convergence. Prior to all statistical analyses, we assessed colinearity (*r* < 0.6) and multicolinearity (variance inflation factor < 4) between independent variables. We ranked the candidate models (same set for each period) based on their AIC values and kept models with ΔAIC < 2.

**Table 2:** Candidate models for caribou use of residual forest patches in Saguenay – Lac-Saint-Jean region, Québec, Canada (2004–2010). Their ranking, using ΔAIC and their weight (*w*_*i*_) are presented for each biological periods. Pearson's *R* correlations between predicted values from best models and real values are also shown as a measure of model fit. See Table 1 for a definition of model variables

Model description	Spring		Calving		Summer		Rut		Winter	
				
	ΔAIC	*w*_*i*_	ΔAIC	*w*_*i*_	ΔAIC	*w*_*i*_	ΔAIC	*w*_*i*_	ΔAIC	*w*_*i*_
Area	4494.11	0.00	5596.99	0.00	18329.81	0.00	9909.81	0.00	30042.43	0.00
Area, Mixed	4087.80	0.00	5578.29	0.00	17974.47	0.00	9449.18	0.00	28513.70	0.00
Area, Cutover, Open, Regen, Coniferous, Wetland	1792.88	0.00	1126.75	0.00	3379.72	0.00	1396.50	0.00	5536.73	0.00
Area, Cutover, Open, Regen, Coniferous, Wetland, Road, Cabin	40.68	0.00	6.19	0.04	179.99	0.00	19.81	0.00	384.51	0.00
Area, Mixed, Road, Cabin	1952.09	0.00	1841.23	0.00	7111.00	0.00	4281.68	0.00	20028.39	0.00
Area, Mixed, Cutover, Open, Regen, Coniferous, Wetland, Road, Cabin	0.00	1.00	0.00	0.96	0.00	1.00	0.00	1.00	0.00	1.00
Best model Pearson's *r* validation	0.826		0.876		0.948		0.881		0.663	

Hurdle models are more efficient to deal with data overdispersion and large number of zeros (patches with no locations inside) than Poisson, Quasi-Poisson or Negative Binomial distributions (Zeileis et al. 2008). These models have two different components: a count data model (Poisson distribution in our case) that is left truncated (at *y* = 1) and a zero hurdle model that is right censored (at *y* = 1) and based on a binomial with logit link. Model parameters *β* are estimated by maximum likelihood. Since patch use could be influenced by period length, location numbers that differ between periods and years, as well as by the ratio of patch size on study area, we added an offset that included all these considerations: log (patch area (ha)/yearly study area (ha) × number of locations in that period). All statistical analyses were conduct with R 2.13.2 (R Core Team 2010).

## Results

### Range of influence

**Figure 2:**
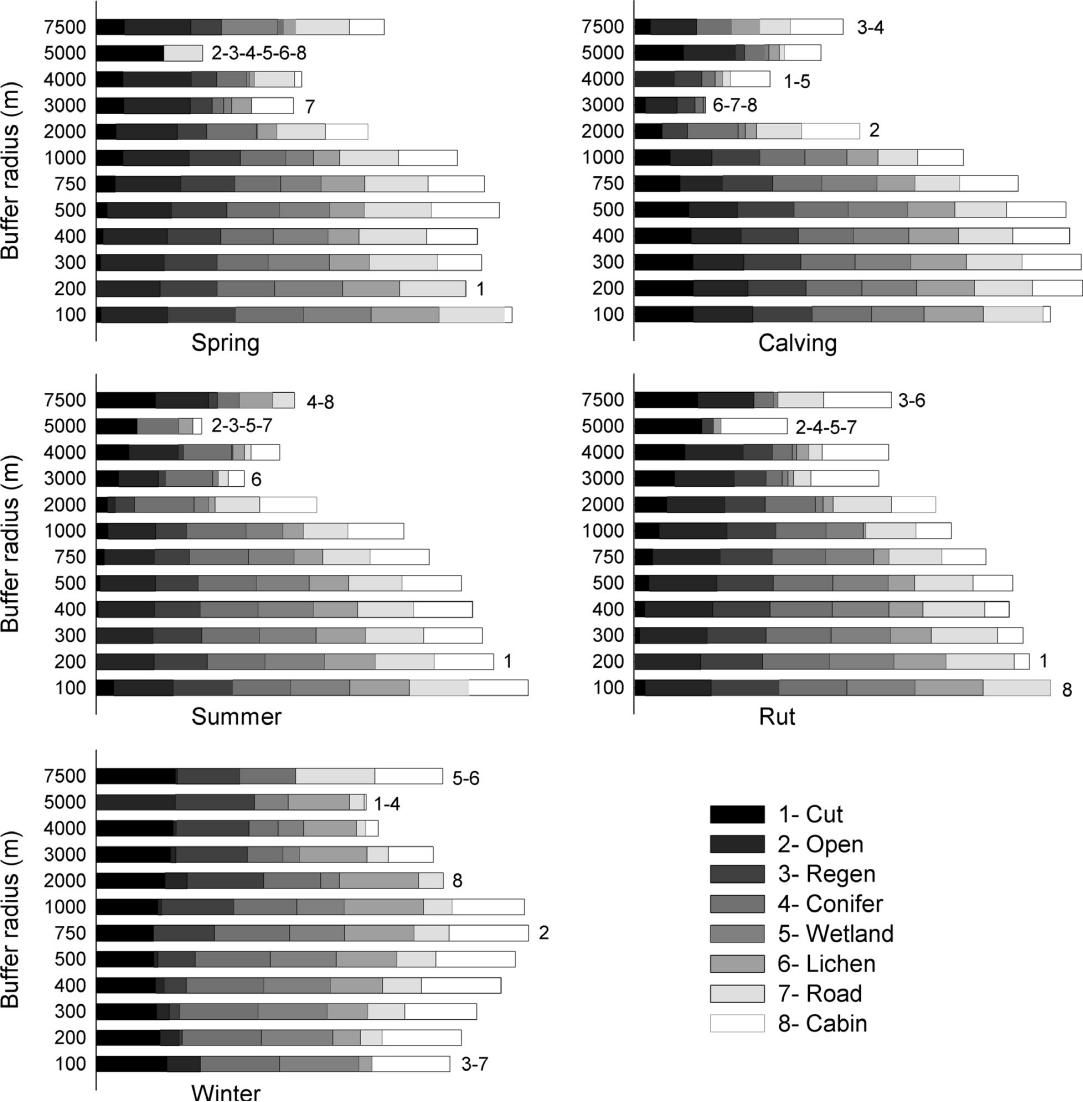
Stacked bar charts representing standardized ΔAIC for each variable within incremental radius buffer sizes (m) for each period. Variable numbers are written beside the radius for which they reach the lower ΔAIC.

Caribou are influenced by their environment at a relatively large scale; the most explicative buffer radii varied from 100 to 7500 m, depending on the period and the variable (Fig. 2), but the vast majority of these variables reach their lower AIC value at radii >2000 m. Cutovers seemed to influence caribou patch use at a smaller scale more often than other variables.

### Model selection and composition

**Figure 3:**
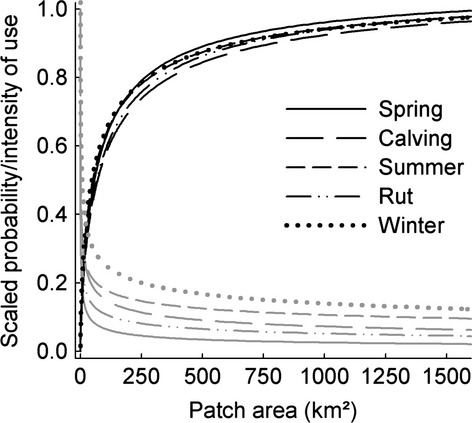
Predicted probability (black lines) by used patch size and predicted scaled intensity of use (no. of locations/km^2^) with increasing patch size (gray line) using the most parsimonious model for each period. All other variables included in models were kept constant at their mean value.

Model 6, that included the whole set of variables (Table 2), always obtained the lowest AIC values among the candidate models and showed strong fit to the data, as the Pearson's *R* between predicted and real values ranged from 0.663 to 0.947, depending on the biological period. The hurdle model is composed of two parts; therefore, we obtained two coefficients for each variable (Table 3). As expected, patch area had a strong positive influence on patch use, both on the probability of a patch of being used and, when it was used, on the abundance of locations in a patch; nevertheless, the relationship between patch area and the density of location in a patch was negative (see Fig. 3). Residual forest patch use by caribou was strongly influenced by the composition of the surrounding environment. Disturbed habitats (such as cutovers and regenerating stands) surrounding a residual patch had negative effects on patch use except during the calving and winter periods (cutovers), as well as spring, calving and rut (regenerating stands) in the Poisson portion of the models (Table 3). Undisturbed habitat types like mature conifer stands and wetlands had an overall negative impact on patch use, while the pattern was less consistent for open lichen woodlands. Perennial disturbances had varying effects depending on their type. Roads had a weak negative influence on caribou presence except during calving, when its effect was stronger, but it had an opposite and positive influence on the intensity of use yearlong. Finally, patches surrounded by a higher density of cabins were avoided or used less intensively by caribou.

**Table 3:** Coefficient estimates (±standard error) of the independent variables of the most parsimonious hurdle models per period, binomial with logit link (Log) and Poisson (Pois) regressions, explaining use of residual forest patches in the Saguenay – Lac-Saint-Jean region, Québec, Canada (2004–2010). See Table 2 for description and ranking of models

Period	Log (Area)		Mixed		Cut		Open		Regen		Conifer		Wetland		Lichen		Road		Cabin	
									
	β	±SE	β	±SE	β	±SE	β	±SE	β	±SE	β	±SE	β	±SE	β	±SE	β	±SE	β	±SE
Spring																				
Log	1.79*	0.12	5.01*	1.13	0.30	0.38	2.34*	1.11	−1.84*	0.63	−1.21	0.82	−10.30*	4.34	1.11	4.11	−0.23	0.14	−1.56*	0.48
Pois	1.35*	0.01	−0.95*	0.19	−1.02*	0.07	−1.92*	0.23	0.94*	0.11	−2.73*	0.16	−22.45*	0.99	8.14*	0.64	0.48*	0.02	−3.18*	0.10
Calving																				
Log	1.66*	0.12	−1.41	1.21	−0.63	0.71	2.89*	0.79	−0.25	0.71	0.66	0.57	−8.67*	4.14	−7.36	4.83	−0.29*	0.13	0.09	0.25
Pois	1.56*	0.01	0.67*	0.22	1.01*	0.10	−1.70*	0.14	0.64*	0.12	−1.57*	0.09	−25.32*	1.19	9.16*	0.87	0.63*	0.02	−0.84*	0.04
Summer																				
Log	1.60*	0.11	−1.65	1.28	−0.51	0.31	−5.67*	1.79	−3.86*	0.58	−0.16	0.71	−31.92*	6.57	6.57	3.77	−0.09†	0.14	1.12*	0.29
Pois	1.77*	0.01	−2.97*	0.23	−1.51*	0.05	0.01	0.19	−0.26*	0.08	−2.51*	0.07	−42.01*	1.04	−2.70*	0.79	0.61*	0.02	−0.99*	0.03
Rut																				
Log	1.73*	0.12	−7.75*	2.17	−0.11	0.35	−1.93	1.44	−3.03*	0.73	−0.58	0.79	−4.11	4.34	2.49	4.24	0.18	0.14	−0.39*	0.18
Pois	1.56*	0.01	−1.12*	0.39	−1.80*	0.06	−2.51*	0.26	0.45*	0.09	−2.78*	0.15	−45.79*	1.09	15.75*	0.74	0.24*	0.02	−0.91*	0.03
Winter																				
Log	1.54*	0.10	−6.96*	1.53	0.98	0.61	1.21*	0.49	−1.56*	0.69	−1.37†	0.72	−14.30*	4.36	−2.48	4.03	−0.06	0.05	−1.05*	0.24
Pois	1.70*	0.01	4.31*	0.22	0.83*	0.07	2.27*	0.04	−1.87*	0.09	−5.03*	0.09	−43.34*	0.63	10.11*	0.40	0.40*	0.01	−0.66*	0.03

* *P* < 0.05;

† 0.05 > *P* < 0.10.

### Model prediction

As patch size was the most manageable characteristic from a management perspective, we modeled its influence on probability of caribou occurrence (binomial with logit link) and intensity of use (Poisson). We obtained opposite patterns (Fig. 3); increasing patch size favored caribou presence, yet intensity (locations/km^2^) decreased sharply between 0.3 and 50 km^2^ and remained constant above 100 km^2^. The probability of use increased incrementally; for example, the patch size needed to reach 50%, 75%, and 95% of probability of use was 72, 270, and 1350 km^2^, respectively, during calving. The resulting patterns of relative probability of occurrence and intensity of use were similar for all biological periods.

## Discussion

Using a multiscale approach, we demonstrated how the composition, size, and surrounding environment of residual forest patches in a heavily managed landscape can influence caribou behavior. These new findings can help orient caribou conservation strategies by providing information on habitat attributes influencing space use behavior of caribou living in forests with timber extraction. Using residual forest patches as the sampling unit rather than individual caribou (Hins et al. 2009) or aerial surveys (Fortin et al. 2008) allowed us to model the use of a given habitat patch, while considering the surrounding environment at a relatively large scale. Although several studies have characterized hierarchical patterns of caribou habitat selection (e.g., Rettie and Messier 2000; Hins et al. 2009; Leclerc et al. 2012), our study was innovative by modeling the occurrence probability and the intensity of use within residual forest patches based on their intrinsic characteristics and the composition and structure of the surrounding matrix; this allowed us to quantify their real contribution to caribou conservation. Although we did not model caribou survival and recruitment within residual patches according to the matrix characteristics, space use and habitat selection patterns expressed by a group of individuals are known to influence demographic trends at the population level (McLoughlin et al. 2005).

### Range of influence

Our results show that the surrounding environment influences caribou space use at a very large scale throughout the year. Therefore, the decision to use a given habitat, even a preferred habitat, is mediated by the amount and the configuration of other habitat features within a buffer up to 7500 m beyond the habitat patch edge. Most variables had a stronger influence at large scale (>2 km) for all periods (Fig. 2), although this pattern was weaker during winter. This trend is likely due to caribou sensitivity to landscape features (Leclerc et al. 2012; DeCesare et al. 2012; Fortin et al. 2013), especially during spring, calving, and summer (Leblond et al. 2011), with summer being critical for calf survival (Dussault et al. 2012). The greater variability in the range of influence during winter may originate from a seasonal diet shift, from forbs and herbs to lichen-based food supply (Bergerud 1972; Klein 1990). Indeed, forage opportunities and vulnerability to predation decreased (at least for calves; Pinard et al. 2012) as winter progressed, forcing caribou to modify their space use and habitat selection patterns, and possibly to use suitable habitat patches even if they are embedded in a less suitable matrix (Smith et al. 2000). Furthermore, female caribou could be more prone to use riskier habitats during winter as their calves are less vulnerable to wolves (Pinard et al. 2012) and black bears are denning at this time (Schooley et al. 1994).

The differential use of a residual patch based on the characteristics of the neighboring environment can also be interpreted as a functional response (Hebblewhite and Merrill 2008; Moreau et al. 2012) because two potentially suitable habitats might not have the same value depending on their configuration at the landscape scale. By considering caribou behavior from the habitat patch perspective, our approach synthesized the intrinsic patch value (i.e., habitat composition and size) with the influence of the landscape matrix.

### Habitat features and perennial disturbances

We demonstrated that the intrinsic residual forest patch composition influenced caribou use. An increasing proportion of deciduous and mixed stands within residual forest patches were found to generally decrease caribou use. The relative avoidance of deciduous and mixed mature forest stands by caribou is frequently common and is usually explained by the increased use of such stands by predators and alternate prey (Dussault et al. 2005; Hins et al. 2009; Lesmerises et al. 2012).

Habitat features surrounding the residual forest patch strongly influence its use by caribou. The large-scale influence (up to 7500 m) of the surrounding environment, especially for variables related to habitat types suitable to alternative prey and predators, is consistent with the hypothesis that predation is the primary factor explaining caribou behavior in managed landscapes (Leclerc et al. 2012). For example, disturbed stands (i.e., regenerating stands, cutovers) suitable for other ungulate species and predators (Dussault et al. 2005; Brodeur et al. 2008; Lesmerises et al. 2012) in the surrounding matrix decreased the probability that a residual forest patch would be occupied by caribou in almost all periods. However, some of our results were inconsistent with this hypothesis; an increased proportion of cutovers around forest patches favored increased caribou location density during calving and winter and regenerating patches had the same effect during spring, calving, and rut. This reversed influence of disturbed habitats (i.e., negative for probability of use, yet positive for intensity of use) during these periods, could be part of the above-mentioned predator-avoidance strategy where caribou strongly avoided patches embedded in disturbed landscape but, when these remnants represent the only preferential habitat available, concentrate their activities within these patches. Such results support the hypothesis of Berryman and Hawkins (2006), who suggested that animals can concentrate themselves in refuges, that is, suitable and low-risk habitats that remain in a disturbed landscape. Indeed, if prior to forest harvesting, caribou were present in a particular area, those individuals may continue to return where they previously had reproductive success or experienced low predation risk (Ferguson and Elkie 2004); this was observed in our study area, even after local increases in human-induced disturbances (Faille et al. 2010).

We found that perennial disturbances (roads and cabins) have important and generally negative influences on caribou occurrence within a patch, which can be related to direct and indirect effects (e.g., encounter, noise, and odors) of human-induced disturbances which trigger antipredator responses (e.g., vigilance, fleeing, habitat selection; Frid and Dill 2002). Here, we report that roads have a negative or null influence on caribou occurrence, but cutovers and regenerating stands promoted caribou concentration in residual forest patches (i.e., refuge effect) when found at high densities in the area surrounding forest patches. Roads also represent an additional threat for caribou as roads are selected by wolves (Whittington et al. 2005; Lesmerises et al. 2012) and could therefore increase predation risk when found near preferential caribou habitat. Cabin density in the surrounding matrix had a negative impact on caribou use of a residual patch during all periods except summer (breeding period for cow–calf pairs), when increased cabin density enhanced occurrence but decreased location density. This mediated association with human activity could be an antipredator strategy within an increasingly disturbed landscape, where traditionally safe habitats are rare and human presence ubiquitous; caribou may be selecting for anthropogenic features that are avoided by predators, a situation previously demonstrated in other predator–prey interactions (e.g., wolf *Canis lupus*–elk *Cervus canadensis*, grizzly bear *Ursus arctos*–moose *Alces alces*; Hebblewhite et al. 2005; Berger 2007).

The amount of mature coniferous forest and wetland habitat in the surrounding matrix had a strong negative influence on caribou use of residual forest patches (both presence and location density) in all periods. This result seems to be in opposition to the well-documented caribou preference for landscapes with low disturbance levels (Fortin et al. 2008). However, rather than being interpreted as a detrimental effect of mature coniferous forests on patch use, we suggest that a residual forest patch embedded in a matrix dominated by preferential caribou habitat may result in a decrease of its relative attractiveness within the landscape. Indeed, the abundance of mature coniferous forests and wetlands in the matrix could favor the dilution of caribou activities outside residual patches, dampening the refuge effect (Sih 1987; Berryman and Hawkins 2006).

### Area influences caribou occurrence and patch use

Residual forest patch area was the most important variable explaining both occurrence and intensity of use, but in different manners. Within continuous forest or large residual forest patches, caribou can express their adaptive dispersal behavior and distribute themselves at very low density (i.e., spacing out strategy; Ferguson and Elkie 2004), thereby increasing search time and lowering success rates for predators (Bergerud and Page 1987). Whereas we showed that caribou typically avoid small forest fragments, some caribou may confine themselves within smaller residual stands offering the only closed-canopy habitat in a heavily disturbed forest matrix (i.e., refuge effect), where predation risk is often higher (Tremblay-Gendron 2012). Such behavioral responses can be exacerbated by the relatively high range fidelity observed in our study area (Faille et al. 2010). However, we think that the concentration of caribou activity in small habitat fragments cannot be sustained for long periods of time and that these small residual forest patches will likely be abandoned by caribou in the near future. Moreover, we believe that this maladaptive habitat selection behavior could result in a “two-step” extirpation process (Kuussaari et al. 2009) following anthropogenic habitat disturbance: (1) caribou are initially confined in numerous small residual forest patches for several years because of site fidelity (Faille et al. 2010) and (2) are thereafter displaced or killed (calves, then older senescent adults) by predators which express a numerical response in the surrounding disturbed matrix (Debinski and Holt 2000; Dussault et al. 2012). We believe that this process could partially explain the spatial (i.e., tolerance threshold of 13 km to nearest cutover) and temporal lags (i.e., two decades between timber harvesting and extirpation) identified by Vors et al. (2007). Moreover, it helps us to clarify the mechanisms underlying recent caribou range recession following past (and in some cases current) forest management strategies, which have resulted in the retention of only small fragmented and isolated forest remnants. Such delays between landscape disturbances and population responses could make more complicated the evaluation of the effectiveness of a recovery strategy, as using small residual forest patches could represent an ecological trap for caribou, that is, the selection of an attractive sink habitat (Delibes et al. 2001; Battin 2004).

### Implications for conservation and management

Our methodological approach could be applied to a wide range of species that live in disturbed habitat matrices. We have illustrated different scales at which animals trade-off limiting factors such as predation and food access in a spatially structured landscape by determining the range of influence that surrounding landscapes have on animal distribution and in a hierarchical manner (i.e., occurrence followed by intensity). Using this methodology could offer conservation authorities relevant knowledge on a variety of species responses to different land protection alternatives, therefore providing scientific support to the decision-making process.

By describing caribou space use in highly managed forests, we showed that this species can be sensitive to human-induced disturbances at the landscape scale. Based on our results, we recommend that residual forest patches must be larger than 100 km^2^ to avoid crowding which may increase caribou vulnerability to predation. Optimally these patches should be much larger in size to ensure their use by caribou (Fig. 3). It could be tempting to manage landscapes to keep only the smaller forest patches, as it is less restrictive for harvest planners. However, we must keep in mind that only a fraction of 100 km^2^ patches are used (57–64% depending on the period; Fig. 3). Our results fill the need outlined in the recent recovery strategy for boreal populations of caribou in Canada (Environment Canada 2012) about the size, composition, and configuration of suitable habitat patches within managed caribou ranges. We explicitly illustrated how detrimental and inefficient it could be to spread human-induced disturbances throughout caribou ranges, then creating a multitude of small patches unused by or unsuitable for caribou, even if the 65% threshold of undisturbed habitat is respected (Environment Canada 2011).

Given that caribou are considered vulnerable or endangered (depending on the jurisdiction), the precautionary principle suggests that managers should try to reach the most conservative size when planning protected residual forests. The significant influence that surrounding matrix features have, up to 7500 m, should also be considered. The long-term effect of cutover presence and the negative, perennial influence of roads and cabins highlight the importance of greatly reducing the disturbance level in regions that surround caribou conservation areas. Our results illustrate how difficult it is for caribou ecologists, forest industry workers, and policy makers to reconcile industry desires with conservation issues, but underline the necessity for an integrative management of boreal forest landscapes.
